# Physical activity, recreational screen time, and depressive symptoms among Chinese children and adolescents: a three-wave cross-lagged study during the COVID-19 pandemic

**DOI:** 10.1186/s13034-024-00705-3

**Published:** 2024-01-19

**Authors:** Yujie Liu, Erliang Zhang, Huilun Li, Xin Ge, Fan Hu, Yong Cai, Mi Xiang

**Affiliations:** 1 Hainan Branch, Shanghai Children’s Medical Center, School of Medicine, Shanghai Jiao Tong University, Sanya, 572022 China; 2 School of Public Health, Shanghai Jiao Tong University, Shanghai, 200025 China; 3 Public Health department, Hongqiao International Institute of Medicine, Tongren Hospital, Shanghai Jiao Tong University School of Medicine, Shanghai, 200025 China

**Keywords:** Children and adolescents, Physical activity, Recreational screen time, Depressive symptoms

## Abstract

**Background:**

Longitudinal evidence is lacking on the interplay between lifestyle behaviors and depressive symptoms, especially in the context of the COVID-19 pandemic. This study investigated the changes in physical activity and recreational screen time during the pandemic, along with their reciprocal associations with depressive symptoms among children and adolescents.

**Methods:**

The public health emergency due to the pandemic started in January 2023 and lasted for two months in Shanghai, China. A three-wave longitudinal study was conducted among 1,666 children and adolescents (6–18 years) in January, March, and July 2023. Moderate-to-vigorous intensity physical activity (MVPA), recreational screen time, and depressive symptoms were measured using self-reported questionnaires. Random-intercept cross-lagged panel models were constructed to examine the bidirectional associations between physical activity and recreational screen time with depressive symptoms.

**Results:**

Children and adolescents experienced a significant decrease in MVPA and a substantial increase in recreational screen time during the pandemic, which failed to return to pre-pandemic levels post-pandemic. Pre-pandemic MVPA was negatively associated with subsequent depressive symptoms (β = -0.147). Conversely, pre-pandemic depressive symptoms were positively associated with subsequent recreational screen time (β = 0.085), which in turn predicted heightened post-pandemic depressive symptoms (β = 0.076). When stratified by age, significant associations were found in adolescents but not children.

**Conclusions:**

Sustained unhealthy changes in physical activity and recreational screen time were observed during the COVID-19 pandemic among children and adolescents. This study elucidates a potential reciprocal relationship between lifestyle behaviors and mental well-being. Effective interventions are emphasized to counter the negative impacts of insufficient physical activity and excessive screen use on the mental health of children and adolescents.

## Background

Depression is among the most common mental disorders that frequently develop during childhood and adolescents [1]. In China, a total of 3.0% of children and adolescents have depressive disorders, with a larger proportion (19.9%) affected by less severe depressive symptoms [2, 3]. The unresolved depressive symptoms in adolescence can persist into adulthood, resulting in poor adult outcomes [4]. To facilitate the timely recognition and intervention of depression in children and adolescents, research has been conducted on the potential benefits of modifiable lifestyle behaviors, such as physical activity and screen time [5–7]. For example, Luo et al. found that meeting the physical activity and recreational screen time guidelines (i.e. physical activity ≥ 60 min/day and recreational screen time ≤ 120 min/day) was associated with a significantly lower risk of depression in Chinese adolescents [8]. Evolving evidence has revealed that participating in physical activity and limiting screen time potentially impact children and adolescents’ mental health [9, 10].

During the COVID-19 outbreak, prolonged time at home and limitation of outdoor activities exerted profound influence on children and adolescents’ life pattern, resulting in significantly less physical activity and more screen time [11]. In addition, previous studies have shown that subsequent to the remission of the pandemic, children and adolescents sustained a reduced level of physical activity and an increased duration of screen use compared to the pre-pandemic level [12, 13]. The unhealthy changes in lifestyle behaviors may exacerbate children and adolescents’ mental health. Several cross-sectional studies during the pandemic have revealed that the decreased physical activity and increased screen time were positively associated with depressive symptoms during the pandemic [14, 15].

Moreover, the associations between lifestyle behaviors and depressive symptoms could be bidirectional [16]. It has been demonstrated that children and adolescents who engage in physical activity and decrease screen time exhibit enhanced mental health well-being and reduced depressive symptoms [9, 17]. Conversely, children and adolescents with suboptimal mental health may also decrease their physical activity engagement and increase their screen-based activities, thereby further exacerbating depressive symptoms [18]. Using cross-lagged panel models, several pre-pandemic studies have found that physical activity and recreational screen time exhibited reciprocal associations with depressive symptoms in adolescents [19, 20]. However, no longitudinal study has been conducted to explore the interplays between physical activity, recreational screen time, and depressive symptoms in the context of the COVID-19 pandemic, when changes in lifestyle behaviors may result in alterations in such bidirectional relationships.

To fill the gap in the current research, we utilized data from a three-wave longitudinal study conducted among children and adolescents in Shanghai, China before, during, and after the COVID-19 pandemic. Throughout the course of this investigation period, we were able to observe how lifestyle behaviors recovered in response to external environmental changes and explore how such lifestyle changes affected the mental well-being of children and adolescents. A random-intercept cross-lagged panel model (RI-CLPM), was used in the present study to separate fluctuating within-person variations from stable between-person traits in the longitudinal data. Specifically, the aim of the present study were (1) to investigate changes in physical activity and recreational screen time across the three stages of the COVID-19 pandemic; (2) to analyze whether changes in physical activity and recreational screen time could predict subsequent changes in depressive symptoms or vice versa within the same person.

## Methods

### Participants

A three-wave longitudinal study was conducted among children and adolescents in Shanghai, China. Seven districts in Shanghai agreed to participate in the survey, and one to two schools were selected from each district via simple random sampling. A large-scale cross-sectional survey was initially conducted in ten schools. Subsequently, five schools were selected out of the ten via simple random sampling for follow-up. One primary school, three junior high schools, and one school integrating primary and junior high schools participated in the follow-up investigations. All the children and adolescents in the selected schools and their parents were invited. There was no significant difference in sociodemographic variables between the students in the retained schools and those excluded. Grade 9 students voluntarily withdrew from the survey because they were preparing for the junior high school graduation exam and were unable to allocate time for participation in the follow-up survey.

**Fig. 1:**
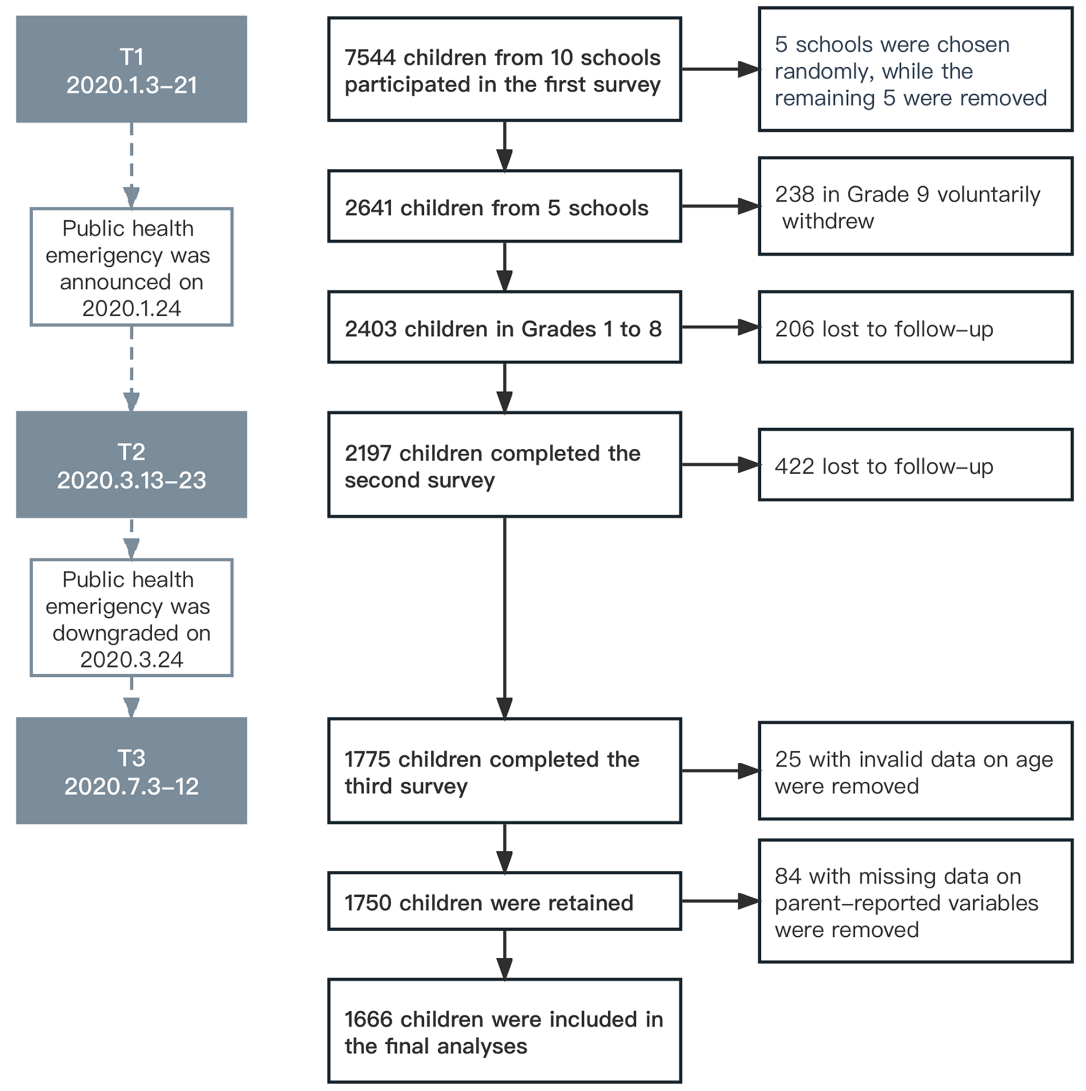
Flow of participants. *T1: Time point 1; T2: Time point 2; T3: Time point 3

The first survey recruited 2,641 children and adolescents in the five schools between January 3rd and 21st, 2020, with 2,403 enrolled in Grades 1 to 8 (Time point 1, T1). Then a public health emergency for the COVID-19 pandemic was announced in Shanghai on January 24th, 2020. The government issued stay-at-home orders and enforced school closures to curb the continued spread of the infection. Children and adolescents were confined to their homes and participated in online classes during this time. Approximately two months after the COVID-19 outbreak, the second survey was conducted among 2,197 children and adolescents between March 13th and 23rd, 2020 (Time point 2, T2), right before the public health emergency response was downgraded from level 1 to level 2 in Shanghai on March 24th, 2020. The lockdown was relaxed and schools began to reopen then. Finally, a total of 1,775 (73.9%) children and adolescents completed the third survey between July 3rd and 12th, 2020 (Time point 3, T3) when the pandemic subsided and life returned to normal. Participants with missing or invalid data on age (*n* = 25), or parent-reported socioeconomic status variables (*n* = 84) were excluded. The final sample consisted of 1,666 children and adolescents. The flow chart of participants in this study is presented in Fig. 1.

The questionnaire link was generated using the online platform Wenjuanxing (www.wjx.cn). Prior to the surveys, school teachers received comprehensive training from the research team to ensure they fully understood the implications and requirements of the study. In each survey, questionnaires were distributed online via WeChat by the head teacher to each parent and their children. To address any questions or uncertainties that arose during the questionnaire survey, we offered continuous online support to assist students and parents. Considering the possible recalling bias and comprehension difficulties in young children, parents of primary school students were instructed to complete the questionnaire together with their children. Once the online questionnaire was filled out, the data was thoroughly examined to confirm its completeness. Written informed consent was obtained from each parent before data collection.

### Measures

The questionnaire consisted of a child/adolescent section and a parent section. Physical activity, screen time, depressive symptoms, sex, age, and grade were derived from the child/adolescent section, while parental educational level and family annual income were derived from the parent section.

#### Moderate-to-vigorous intensity physical activity (MVPA)

MVPA was measured using the Global Physical Activity Questionnaire (GPAQ) which was developed by the World Health Organization [21] and modified for use in children and adolescents in previous studies [22, 23]. The GPAQ consists of 16 questions that assess physical activity participation in different settings. Multiple questions about intensity (moderate or vigorous), frequency (days in a typical week), and duration (hours and minutes on a typical day) were used to calculate the daily amount of time spent on MVPA participation. According to the 24-Hour Movement Guidelines, children and adolescents should have at least 60 min/per day of MVPA [24].

#### Recreational screen time

Recreational screen time was reported using the number of days per week and the time spent per day on (1) watching TV/videos, VCDs, and DVDs; (2) computer/smartphone gaming; (3) social media use (QQ, WeChat, etc.); and (4) browsing webpages (news, douban, etc.). Screen use items were built on the different categories of screen use proposed by previous studies [25] as well as results derived from our pilot study about screen use, including recreational and educational items. This study exclusively focused on the health effects associated with recreational screen time, following the 24-Hour Movement Guidelines, which recommend a maximum of 2 h of daily recreational screen time for children and adolescents [24]. This ensures that our investigation aligns closely with these established recommendations. The daily recreational screen time was calculated by averaging the amount of time spent on weekdays and weekends.

#### Depressive symptoms

Depressive symptoms were measured using the Children Depression Inventory-Short Form (CDI-S) [26]. The CDI-S consists of 10 items, and each item requires respondents to rate the severity of different depressive symptoms (e.g., “I am sad once in a while”/ “I am sad many times”/ “I am sad all the time”) with a score of 0–2 [27]. The summed scores yield a global score ranging from 0 to 20, with higher scores indicating a higher level of depressive symptoms. A cut-off score of 7 and greater was recommended to indicate depressive symptoms in Chinese children and adolescents [28]. The Chinese version of the CDI-S has shown good internal consistency: the Cronbach’s alpha coefficient ranges from 0.72 to 0.75 for children [29, 30] and from 0.86 to 0.90 for adolescents [31, 32].

#### Demographic covariates

Demographic covariates included sex (boy; girl), age, grade (1–3; 4–6; 7–9), mother’s and father’s educational level (junior high school and below; senior high school; college/university; master/doctor), and family annual income (< 100,000¥, 100,000-200,000¥, 200,000-400,000¥, and > 400,000¥.

### Statistical analysis

First, we described the MVPA, recreational screen time, and depressive symptoms using means and standard deviations (SD). Proportions of children and adolescents who met the guidelines of MVPA and recreational screen time as well as the CDI-S cut-off were described using numbers and frequencies. Then mixed-effects models were fitted to examine the changes in MVPA and recreational screen time across the three surveys. When significant main effects of time were detected, Bonferroni-corrected pairwise comparisons were performed as post hoc tests to explore differences between the measurements.

RI-CLPMs were constructed to assess the longitudinal associations between MVPA, recreational screen time, and depressive symptoms. In the RI-CLPMs, random intercepts were defined with latent variables using repeated measures as indicators, with factor loadings constrained to 1. The within-person components were created by regressing the observed variables for each measurement on its own latent factor. Autoregressive and cross-lagged associations between the within-person components were then specified and freely estimated. In this study, the hypothetic associations between MVPA, recreational screen time, and depressive symptoms were explored separately in two RI-CLPMs. Sex, age, parent’s education level, and family annual income were controlled in the model by including time-invariant predictors for the random intercepts.

The RI-CLPMs were fitted using the Lavaan package in R [33], with the robust full information maximum likelihood (FIML) to handle missing data on MVPA, recreational screen time, and depressive symptoms. The goodness-of-fit of the model was assessed by a series of fit indices (comparative fit index [CFI], Tucker–Lewis index [TLI], root mean square error of approximation [RMSEA], standardized root mean square residual [SRMR]): CFI and TLI > 0.90 for acceptable and > 0.95 for good fit, RMSEA, SRMR < 0.08 for good fit [34, 35]. *P*-values < 0.05 were considered statistically significant.

### Ethics

All procedures performed in studies involving human participants were in accordance with the 1964 Helsinki declaration. The study protocol was approved by the Ethics Committee of Shanghai Jiao Tong University School of Medicine (SJUPN-201,813).

## Results

### Descriptive statistics and bivariate correlation

Of the included 1,666 children and adolescents, 860 (51.6%) were boys and 806 (48.3%) were girls, ranging in age from 6 to 16 years (mean age [SD]: 10.6 [2.31] years). Descriptive statistics of baseline sample characteristics of the participants are presented in Table 1.

**Table 1:** Characteristics of participants at baseline

	Total (*n* = 1666)	Boy (*n* = 860)	Girl (*n* = 806)
Age, mean ± SD	10.6 ± 2.31	10.6 ± 2.33	10.6 ± 2.29
Child (< 10 years old)	524 (31.5%)	281 (32.7%)	243 (30.1%)
Adolescent (≥ 10 years old)	1142 (68.5%)	579 (67.3%)	563 (69.9%)
Mother’s education level, n (%)			
Middle school or below	157 (9.4%)	74 (8.6%)	83 (10.3%)
High or vocational school	327 (19.6%)	161 (18.7%)	166 (20.6%)
College or university	1099 (66.0%)	577 (67.1%)	522 (64.8%)
Master or PhD	83 (5.0%)	48 (5.6%)	35 (4.3%)
Father’s education level, n (%)			
Middle school or below	117 (7.0%)	62 (7.2%)	55 (6.8%)
High or vocational school	375 (22.5%)	178 (20.7%)	197 (24.4%)
College or university	1022 (61.3%)	537 (62.4%)	485 (60.2%)
Master or PhD	152 (9.1%)	83 (9.7%)	69 (8.6%)
Household income, n (%)			
<100,000 yuan	183 (11.0%)	87 (10.1%)	96 (11.9%)
100,000-200,000 yuan	491 (29.5%)	252 (29.3%)	239 (29.7%)
200,000-400,000 yuan	578 (34.7%)	302 (35.1%)	276 (34.2%)
>400,000 yuan	277 (16.6%)	142 (16.5%)	135 (16.7%)
Refuse to answer	137 (8.2%)	77 (9.0%)	60 (7.4%)

Abbreviation: SD: Standard deviation

Table 2 shows the means and SDs of daily MVPA recreational screen time, and CDI-S scores across the three time points. The proportions of children and adolescents who met the guidelines of MVPA and recreational screen time and the cut-off of CDI-S scores are also presented in Table 2.

**Table 2:** Descriptive statistics of physical activity, screen time, and depressive symptoms

	T1		T2		T3	
	Descriptive statistics	n	Descriptive statistics	n	Descriptive statistics	n
Physical activity						
Daily MVPA minutes, mean (SD)	79.1 (85.7)	1666	25.7 (64.6)	1665^*^	60.9 (78.9)	1665^*^
Daily MVPA ≥ 1 h, n (%)	769 (46.2%)		192 (11.5%)		583 (34.0%)	
Daily MVPA < 1 h, n (%)	897 (53.8%)		1473 (88.5%)		1082 (65.0%)	
Screen time						
Daily RST minutes, mean (SD)	43.3 (64.0)	1666	111.3 (136.0)	1666	63.4 (85.1)	1665^*^
Daily RST ≤ 2 h, n (%)	1534 (92.1%)		1144 (68.7%)		1416 (85.0%)	
Daily RST > 2 h, n (%)	132 (7.9%)		522 (31.3%)		249 (15.0%)	
Depressive symptoms						
Total CDI-S scores, mean (SD)	4.05 (2.67)	1666	3.80 (2.47)	1666	3.95 (2.72)	1665^*^
CDI-S scores < 7, n (%)	1429 (85.8%)		1457 (87.5%)		1424 (85.5%)	
CDI-S scores ≥ 7, n (%)	237 (14.2%)		209 (12.5%)		242 (14.5%)	

Abbreviation: MVPA: Moderate-to-vigorous intensity physical activity; RST: Recreational screen time; CDI-S: Children Depression Inventory-Short Form; SD: Standard deviation; T1: Time point 1; T2: Time point 2; T3: Time point 3

^*^there is 1 missing value for physical activity at T2, and 1 each for physical activity, screen time, and depressive symptoms at T3

### Changes in MVPA and recreational screen time

**Fig. 2:**
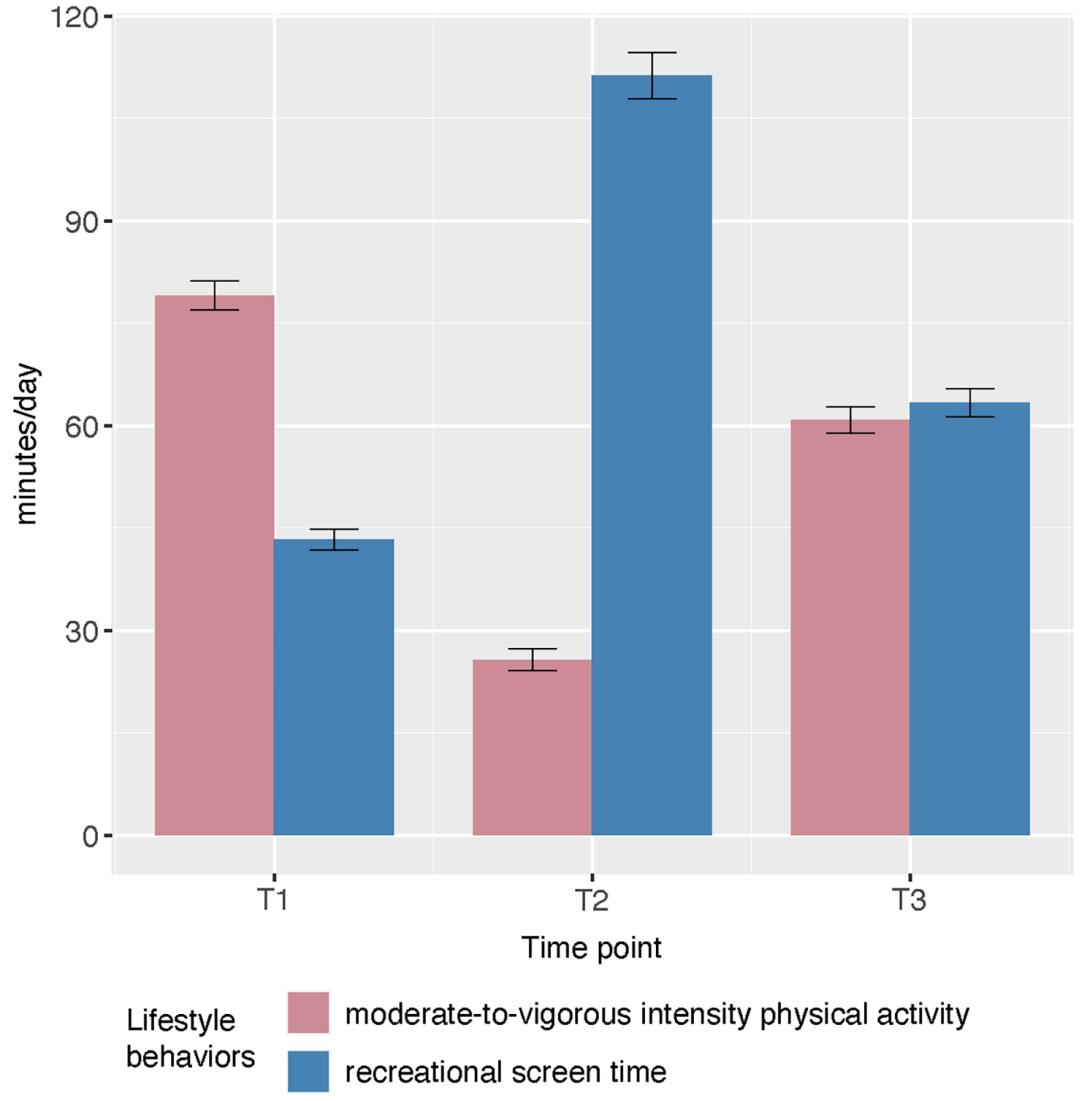
Changes in moderate-to-vigorous intensity physical activity and recreational screen time among children and adolescents. *T1: Time point 1; T2: Time point 2; T3: Time point 3

Figure 2 depicts the changes in the repeated measurements of MVPA and recreational screen time. The mixed-effect model revealed a significant main effect of time on both MVPA (*p* < 0.001) and recreational screen time (*p* < 0.001). According to the results of the post-hoc comparisons, MVPA levels showed a significant decrease from T1 to T2 (Difference_2 − 1_ = -374, *p* < 0.001), with a subsequent increase from T2 to T3 (Difference_3 − 2_ = 246, *p* < 0.001), but remained significantly lower than the baseline level (Difference_3 − 1_ = -128, *p* < 0.001). Conversely, the follow-up period saw a significant increase in recreational screen time from T1 to T2 (Difference_2 − 1_ = 476, *p* < 0.001), which was then followed by a decline from T2 to T3 (Difference_3 − 2_ = -334, *p* < 0.001). However, the recreational screen time at T3 was significantly higher than that of the baseline (Difference_3 − 1_ = 142, *p* < 0.001).

### Longitudinal associations in the RI-CLPM

**Fig. 3:**
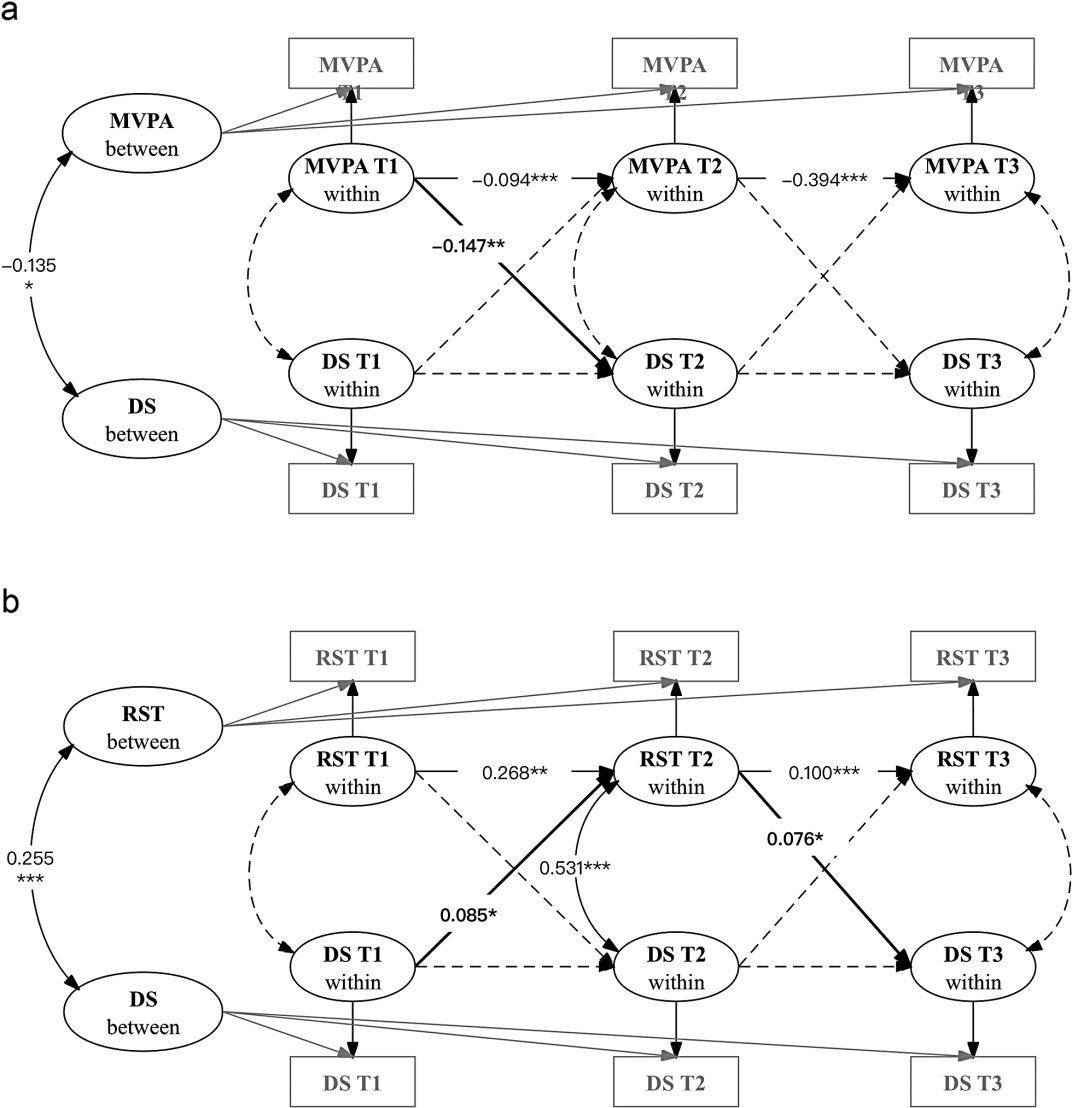
RI-CLPMs of physical activity (2a), screen time (2b), and depressive symptoms *MVPA: Moderate-to-vigorous intensity physical activity; RST: Recreational Screen time; DS: Depressive symptoms; T1: Time point 1; T2: Time point 2; T3: Time point 3

Longitudinal associations among MVPA, recreational screen time, and depressive symptoms are presented in Fig. 3a, b; Table 3. In the RI-CLPMs, the variance was significant for each random intercept (*p* < 0.001), indicating that there were stable, trait-like differences between individuals in MVPA, recreational screen time, and depressive symptoms. At the between level, depressive symptoms were positively associated with recreational screen time (*r* = 0.255, *p* < 0.001) and negatively associated with MVPA (*r* = -0.135, *p* = 0.014). Thus, individuals who reported more recreational screen time or less MVPA across the three measurements had a higher level of depressive symptoms overall.

**Table 3:** Parameter estimates from the RI-CLPMs for physical activity, screen time, and depressive symptoms

	Total		Child (< 10 years)		Adolescents (≥ 10 years)	
	β (SE)	p *value*	β (SE)	p *value*	β (SE)	p *value*
Model 1						
Within-person						
Autoregressive						
MVPA T1 → MVPA T2	-0.094 (0.024)	< 0.001	-0.125 (0.026)	< 0.001	-0.063 (0.032)	0.051
MVPA T2 → MVPA T3	-0.394 (0.065)	< 0.001	-1.009 (0.286)	< 0.001	-0.312 (0.062)	< 0.001
DS T1 → DS T2	-0.016 (0.046)	0.734	0.055 (0.081)	0.498	-0.036 (0.057)	0.526
DS T2 → DS T3	0.077 (0.056)	0.164	-0.022 (0.090)	0.810	0.118 (0.069)	0.086
Cross-lagged						
MVPA T1 → DS T2	-0.147 (0.052)	0.005	-0.064 (0.079)	0.421	-0.181 (0.064)	0.005
MVPA T2 → DS T3	0.108 (0.099)	0.275	0.240 (0.349)	0.491	0.106 (0.102)	0.297
DS T1 → MVPA T2	0.022 (0.018)	0.221	0.016 (0.023)	0.487	0.025 (0.023)	0.288
DS T2 → MVPA T3	0.005 (0.028)	0.853	0.081 (0.059)	0.170	-0.021 (0.033)	0.520
Covariance						
MVPA and DS T1	-0.070 (0.082)	0.392	-0.048 (0.110)	0.661	-0.094 (0.109)	0.390
Residuals MVPA and DS T2	-0.063 (0.078)	0.420	0.059 (0.077)	0.444	-0.115 (0.110)	0.294
Residuals MVPA and DS T3	0.101 (0.081)	0.212	0.049 (0.136)	0.720	0.154 (0.099)	0.119
Between-person						
Covariance (MVPA, DS)	-0.135 (0.055)	0.014	-0.145 (0.059)	0.013	-0.126 (0.074)	0.089
Model 2						
Within-person						
Autoregressive						
RST T1 → RST T2	0.268 (0.099)	0.007	-0.164 (0.166)	0.324	0.405 (0.120)	0.001
RST T2 → RST T3	0.100 (0.017)	< 0.001	0.018 (0.028)	0.516	0.118 (0.021)	< 0.001
DS T1 → DS T2	-0.007 (0.046)	0.887	0.060 (0.080)	0.453	-0.020 (0.057)	0.722
DS T2 → DS T3	0.071 (0.056)	0.205	-0.027 (0.093)	0.767	0.117 (0.069)	0.087
Cross-lagged						
RST T1 → DS T2	-0.103 (0.110)	0.351	-0.027 (0.179)	0.881	-0.127 (0.138)	0.355
RST T2 → DS T3	0.076 (0.032)	0.018	0.060 (0.063)	0.338	0.077 (0.038)	0.041
DS T1 → RST T2	0.085 (0.027)	0.020	0.004 (0.063)	0.950	0.100 (0.044)	0.024
DS T2 → RST T3	-0.003 (0.026)	0.904	0.039 (0.035)	0.265	-0.007 (0.034)	0.838
Covariance						
RST and DS T1	0.071 (0.066)	0.286	-0.010 (0.074)	0.897	0.108 (0.094)	0.249
Residuals RST and DS T2	0.531 (0.137)	< 0.001	0.635 (0.171)	< 0.001	0.529 (0.186)	0.004
Residuals RST and DS T3	0.127 (0.075)	0.092	0.215 (0.088)	0.015	0.087 (0.104)	0.404
Between-person						
Covariance (RST, DS)	0.255 (0.062)	< 0.001	0.033 (0.063)	0.608	0.355 (0.090)	< 0.001

Abbreviation: MVPA: Moderate-to-vigorous intensity physical activity; RST: Recreational screen time; DS: Depressive symptoms; β: estimated standardized beta values; SE: Standard error; T1: Time point 1; T2: Time point 2; T3: Time point 3

As shown in Fig. 3a, b; Table 3, MVPA at T1 predicted subsequent depressive symptoms at T2 at the within level (β = -0.147, *p* = 0.004), indicating that person-centered deviations in MVPA level predicted person-centered changes in depressive symptoms over time. In contrast, depressive symptoms at T1 were associated with increased recreational screen time at T2 (β = 0.085, *p* = 0.020), which in turn predicted elevated depressive symptoms at T3 (β = 0.076, *p* = 0.018). When stratified by age (child < 10 years old and adolescent ≥ 10 years old), the associations of MVPA with depressive symptoms were only observed in the adolescent group (β = -0.181, *p* = 0.005). Similarly, among adolescents, depressive symptoms at T1 were associated with higher recreational screen time at T2 (β = 0.100, *p* = 0.024), which subsequently predicted elevated depressive symptoms at T3 (β = 0.077, *p* = 0.041). However, these associations were insignificant in the child group (Table 3).

The RI-CLPMs showed acceptable goodness of fit (Table 4): CFI = 0.964, TIL = 0.924, AIC = 38990.850, BIC = 39185.905, RMSEA (95%CI) = 0.038 (0.029–0.048), SRMR = 0.020 (Model 1); CFI = 0.972, TIL = 0.941, AIC = 40360.592, BIC = 40555.646, RMSEA (95%CI) = 0.038 (0.028–0.048), SRMR = 0.021 (Model 2).

**Table 4:** Fit indices of the RI-CLPMs

	Model 1	Model 2
CFI	0.964	0.972
TIL	0.924	0.941
AIC	38990.850	40360.592
BIC	39185.905	40555.646
RMSEA (95%CI)	0.038 (0.029–0.048)	0.038 (0.028–0.048)
SRMR	0.020	0.021

Abbreviation: CFI: comparative fit index; TLI: Tucker–Lewis index; AIC: Akaike information criterion; BIC: Bayesian Information Criterion; RMSEA: root mean square error of approximation; SRMR: standardized root mean square residual

## Discussion

Using longitudinal data collected from children and adolescents in Shanghai, China throughout the COVID-19 outbreak, this study sought to investigate the changes in MVPA and recreational screen time and to examine their association with depressive symptoms. To the best of our knowledge, this is the first study to elucidate the within-person temporal dynamics of the association among children and adolescents while controlling for trait-like, time-invariant effects. In this study, we found a significant decline in MVPA levels and a significant increase in recreational screen time during the pandemic, with neither behavior returning to pre-pandemic levels after the remission of the pandemic. Within-person results indicate that MVPA had a negative prospective effect on depressive symptoms, while recreational screen time had a positive reciprocal association with depressive symptoms.

This three-wave longitudinal study extended previous research on lifestyle changes in children and adolescents during the pandemic [36, 37] and provides a clearer understanding of the tremendous negative impact of the COVID-19 pandemic. During the height of the pandemic when social distancing was enforced, numerous restrictions including school closures, mandated home quarantining, and cancellation of extracurricular activities resulted in decreased physical activity and increased recreational screen time. In addition, our results indicate that even after the lifting of restrictions, children and adolescents were unable to return to their normal pattern of lifestyle behaviors. These results align with a previous finding that while the high increase during lockdowns was not sustained, children’s recreational screen time remained higher after schools reopened compared to pre-pandemic levels [38]. Maltagliati et al. suggest that habits altered during lockdown could foster engagement in renewed physical activity behaviors after a context change [39]. Given the negative effects of even short-term reductions in physical activity and increases in screen time [40], it is essential to counteract the detrimental lifestyle changes among children and adolescents.

A significant association was found between MVPA before the COVID-19 outbreak and depressive symptoms during the pandemic, suggesting that children and adolescents who are less physically active relative to their own average levels were more likely to demonstrate higher than usual depressive symptoms. Conversely, there were no significant effects of depressive symptoms on subsequent MVPA at either stage of the pandemic. These findings extend previous cross-sectional studies conducted during isolation in the pandemic [14, 41] and provide insight into the directionality of the relationship between physical activity and depressive symptoms. The health-promoting effect of physical activity is well-recognized and several mechanisms have been proposed to explain this relationship [42], such as enhanced social interaction, self-efficacy, and perceived competence in body image [43]. The present study, which was conducted in a special period with dramatic lifestyle changes, provides further evidence that physical activity has the potential to protect the mental health of children and adolescents from the adverse impact of life-changing events.

Building upon pre-pandemic findings [44], the present study investigated the reciprocal effects of depressive symptoms and recreational screen time during different stages of the COVID-19 pandemic. Results from the RI-CLPM revealed that depressive symptoms before the COVID-19 outbreak were positively associated with recreational screen time during the pandemic, which in turn positively predicted depressive symptoms after the pandemic. The identified timing aligns with previous research [45], suggesting a potential link between increased screen-based activities among children and adolescents with depressive symptoms during outbreaks. This relationship may be indicative of a coping strategy adopted by these individuals to avoid real-world concerns [46]. While this may be an effective short-term solution to psychological distress, it is possible that excessive behavior might eventually become addictive and counterproductive if it continues later in life, potentially leading to higher levels of depressive symptoms after the remission of the pandemic [47]. Such avoidant emotion regulation through recreational screen use could thus form a vicious cycle that continues to exacerbate depressive symptoms rather than alleviate them in the long term [48].

Another finding of our study was that associations between lifestyle behaviors and depressive symptoms varied by age. This pattern aligns with earlier research which indicates that promoting physical activity and limiting screen time have greater benefits in adolescents than in children [49, 50]. As age increases, mental health problems tend to become more prominent [51]. This trend is consistently reflected in our current study, where the prevalence rates of depressive symptoms were much higher among adolescents (12.1-17.1%) compared to children (8.0-10.3%). Consequently, adolescents faced an increased risk of heightened depressive symptoms due to environmental changes amid the COVID-19 pandemic, highlighting the importance of addressing lifestyle impact on this emotionally vulnerable group. Additionally, the association between depressive symptoms and certain lifestyle behaviors, such as excessive recreational screen time, appears to be reciprocal. Therefore, proactive prevention measures are necessary among adolescents to break this vicious cycle as early as possible to mitigate potential long-term consequences.

### Strengths and limitations

To the best of our knowledge, this is the first attempt to explore both the within-person and between-person associations between depressive symptoms and physical activity and recreational screen time in children and adolescents throughout the COVID-19 pandemic. One key strength of this study is the special data collection timing, which coincided with different evolution phases of the pandemic. Through repeated measurements of both exposures and outcomes, we were able to track the developmental patterns of lifestyle behaviors and investigate the direction of their associations with depressive symptoms using RI-CLPMs.

However, there are several limitations that should be acknowledged. First, despite the large sample size, our sample is limited to children and adolescents in Shanghai, China. We should be cautious in generalizing the present findings to nationwide conclusions. Second, the relatively short follow-up time limits our inference on the long-term developmental trend of lifestyle behaviors as well as their complex relationship with depressive symptoms, which may become more pronounced with a longer period. Despite the statistical non-significance observed in our study, further investigation is warranted on the potential long-term effects of depressive symptoms on physical activity. Third, our data on physical activity and recreational screen time are self-reported, which may suffer from inherent recalling bias or social desirability bias. Future studies may benefit from using objective measures to investigate physical activity and recreational screen time (e.g., accelerometer) and to compare results with self-reported data with regard to depressive symptoms. Fourth, the potential impact of non-linear effects may warrant further consideration. While our model primarily focuses on linear relationships, it is essential to recognize that the relationship between lifestyle behaviors and depressive symptoms could involve non-linear dynamics. Finally, although time-invariant confounders were controlled in the RI-CLPMs, the modeling approach did not address the potential effects of other time-varying confounders (e.g., parental health conditions). Therefore, we were not able to make strong causal inferences. Experimental studies are needed to examine the relationship between physical activity, recreational screen time, and subsequent depressive symptoms.

## Conclusion

This longitudinal study established the association between depressive symptoms and two lifestyle behaviors, namely physical activity and recreational screen time, among children and adolescents in three phases of the COVID-19 pandemic. A significant increase in MVPA and a concomitant decline in recreational screen time were observed during the COVID-19 outbreak, which failed to revert to their pre-pandemic levels even after the remission of the pandemic. The persistent unhealthy changes in lifestyle behaviors resulted in adverse effects on individual mental health. In this study, increased MVPA before the COVID-19 outbreak was associated with subsequent lower levels of depressive symptoms. Higher levels of depressive symptoms before the COVID-19 outbreak were associated with subsequent increased recreational screen time, which in turn predicted higher levels of depressive symptoms after the pandemic. These results highlight the complex nature of the relationship between lifestyle behaviors and mental health among children and adolescents, providing support for the benefits of promoting physical activity and limiting recreational screen time as preventive measures against depressive symptoms when experiencing external environmental changes.

## Data Availability

The datasets generated during and/or analyzed during the current study are not publicly available due to privacy or ethical restriction but are available from the corresponding author on reasonable request.
