# Correction: Physical activity, recreational screen time, and depressive symptoms among Chinese children and adolescents: a three-wave crosslagged study during the COVID-19 pandemic

**DOI:** 10.1186/s13034-024-00737-9

**Published:** 2024-03-30

**Authors:** Yujie Liu, Erliang Zhang, Huilun Li, Xin Ge, Fan Hu, Yong Cai, Mi Xiang

**Affiliations:** 1 Hainan Branch, Shanghai Children’s Medical Center, School of Medicine, Shanghai Jiao Tong University, Sanya, 572022 China; 2 School of Public Health, Shanghai Jiao Tong University, Shanghai, 200025 China; 3 Public Health department, Hongqiao International Institute of Medicine, Tongren Hospital, Shanghai Jiao Tong University School of Medicine, Shanghai, 200025 China

**Correction to: Child and Adolescent Psychiatry and Mental Health (2024)**

10.1186/s13034-024-00705-3

Following publication of the original article [1], the author noticed the errors in abstract section and in the value of table 3.

In Abstract under Methods heading, the paragraph should read, “The public health emergency due to the pandemic started in January 2020 and lasted for two months in Shanghai, China. A three-wave longitudinal study was conducted among 1,666 children and adolescents (6–16 years) in January, March, and July 2020. Moderate-to-vigorous intensity physical activity (MVPA), recreational screen time, and depressive symptoms were measured using self-reported questionnaires. Random-intercept cross-lagged panel models were constructed to examine the bidirectional associations between physical activity and recreational screen time with depressive symptoms” instead of “The public health emergency due to the pandemic started in January 2023 and lasted for two months in Shanghai, China. A three-wave longitudinal study was conducted among 1,666 children and adolescents (6–18 years) in January, March, and July 2023. Moderate-to-vigorous intensity physical activity (MVPA), recreational screen time, and depressive symptoms were measured using self-reported questionnaires. Random-intercept cross-lagged panel models were constructed to examine the bidirectional associations between physical activity and recreational screen time with depressive symptoms”.

In Table 3 under the Total column, the p-value corresponding to the “MVPA T1 → DS T2” pathway under the “Cross-lagged” heading should be corrected from 0.005 to 0.004.

